# Analysis of Implant Osseointegration, Bone Repair, and Sinus Mucosa Integrity Using Bio-Oss^®^ and Hyaluronic Acid-Polynucleotide Gel (Regenfast^®^) in Maxillary Sinus Augmentation in Rabbits

**DOI:** 10.3390/dj13070293

**Published:** 2025-06-28

**Authors:** Hiroyuki Omori, Daniele Botticelli, Erick Ricardo Silva, Samuel Porfirio Xavier, Sérgio Luis Scombatti de Souza, Kaoru Kusano, Shunsuke Baba

**Affiliations:** 1 Department of Oral Implantology, School of Dentistry, Osaka Dental University, 8-1 Kuzuhahanazonocho, Hirakata 573-1121, Japan; last_samurai_1206@me.com (H.O.); kusano-k@cc.osaka-dent.ac.jp (K.K.); baba-s@cc.osaka-dent.ac.jp (S.B.); 2 Ardec Academy, 47923 Rimini, Italy; daniele.botticelli@gmail.com; 3 Department of Oral and Maxillofacial Surgery and Periodontics, Faculty of Dentistry of Ribeirão Preto, University of São Paulo, Av. do Café-Subsetor Oeste 11 (N-11), Ribeirão Preto 14040-904, Brazil; erick.silva@usp.br (E.R.S.); scombatti@forp.usp.br (S.L.S.d.S.)

**Keywords:** animal study, bone healing, histology, osseointegration, dental implants, sinus augmentation, sinus membrane perforation, hyaluronic acid, resorbable polynucleotides, xenogeneic bone substitute, osteoinduction

## Abstract

**Background:** The combination of polynucleotides and hyaluronic acid with bovine bone grafts in maxillary sinus lift procedures appears to be a promising strategy to enhance bone regeneration. This study aimed to analyze implant osseointegration, bone repair and sinus mucosa integrity using Bio-Oss^®^ and Hyaluronic Acid-Polynucleotide Gel (Regenfast^®^) in maxillary sinus augmentation in rabbits. **Methods:** Sinus floor elevation was performed in 12 rabbits, with one implant placed per sinus simultaneously. In the control group, sinuses were grafted with deproteinized bovine bone mineral (Bio-Oss^®^) alone; in the test group, Bio-Oss^®^ was combined with Regenfast^®^. Two histological slides were obtained per sinus after 2 weeks (six animals) and 10 weeks (six animals): one from the grafted area alone (non-implant sites), and one from the implant site. Primary outcome variables included the percentage of newly formed bone, the extent of implant osseointegration, and the number of sinus mucosa perforations caused by contact with graft granules. **Results:** After 10 weeks of healing, the test group showed a significantly higher percentage of new bone formation (37.2 ± 6.7%) compared to the control group (26.8 ± 10.0%; *p* = 0.031); osseointegration extended to the implant apex in both groups; fewer sinus mucosa perforations were observed in the test group (*n* = 5) than in the control group (*n* = 14). **Conclusions:** The addition of Regenfast^®^ to Bio-Oss^®^ granules promoted enhanced bone regeneration within the elevated sinus area and was associated with a lower incidence of sinus membrane perforations compared to the use of Bio-Oss^®^ alone.

## 1. Introduction

Dental implants are integral to oral rehabilitation, offering a reliable alternative to conventional prostheses by restoring function and aesthetics. However, implant placement in the posterior maxilla presents challenges due to alveolar resorption and the anatomical characteristics of the maxillary sinus [1]. Bone loss, exacerbated by sinus expansion, often necessitates advanced surgical techniques to enable implant installation.

Sinus floor elevation is a well-established procedure for increasing bone volume in the posterior maxilla, demonstrating high success rates [2]. Nevertheless, maxillary sinus re-pneumatization following sinus mucosa elevation has been frequently described [3,4,5,6,7,8]. To enhance osseointegration and procedural predictability, various biomaterials have been employed to stabilize the sinus floor [9].

Among these materials, xenogeneic bone grafts have been extensively investigated. Bio-Oss^®^, processed at low temperatures (300 °C), is frequently utilized in both clinical [10,11,12,13,14,15] and preclinical research [3,4,16,17,18]. Bio-Oss^®^ serves as an osteoconductive scaffold, supporting bone formation while maintaining graft volume due to its slow resorption rate [3,4,16,17]. Its biocompatibility and effectiveness have been well documented [19,20,21].

Recent strategies have focused on optimizing tissue repair mechanisms to improve clinical outcomes, particularly through the modulation of fibroblast activity [22]. This approach is especially relevant in compromised tissue healing scenarios. Polynucleotides, high-molecular-weight polymers composed of purified deoxyribonucleotide chains, have shown regenerative potential by promoting fibroblast proliferation, protein synthesis, and collagen production [23]. Since 2004, polynucleotides have been incorporated into Class III medical devices for wound healing and intra-articular applications due to their viscoelastic properties [22,24]. They are also utilized in dermatology and aesthetic medicine to enhance skin hydration and elasticity [25].

Hyaluronic acid, a glycosaminoglycan found in connective tissues, plays a crucial role in organogenesis, cell migration, and tissue repair [26,27,28,29]. Hyaluronic acid is biodegradable and bioresorbable, facilitating tissue lubrication, cell differentiation, and wound healing [30]. In dentistry, hyaluronic acid has been explored for its potential in periodontal regeneration, as it constitutes a key component of the periodontal ligament matrix and influences cell adhesion, migration, and differentiation [31,32,33]. Clinical studies have shown its efficacy in reducing bleeding on probing and probing depths in patients with chronic periodontitis [34,35].

A recent study by our group [36], which reported other applications of hyaluronic acid in dentistry, evaluated the combination of polynucleotides and hyaluronic acid with Bio-Oss^®^ granules for sinus augmentation in rabbits. Histological analyses at 2 and 10 weeks revealed no significant differences in bone formation between test and control sites, with no indication of enhanced regeneration or mitigation of adverse effects related to sinus mucosa perforations.

Building upon these findings, the present study aims to assess the impact of hyaluronic acid and polynucleotide gel on bone formation and implant osseointegration through histological analysis. This is the first study to investigate the application of this gel in conjunction with implants in a maxillary sinus augmentation model. Based on prior results, we hypothesize that the hyaluronic acid and polynucleotide gel combination, with xenograft material, may have a limited or null effect on bone formation and osseointegration, though the precise outcome remains to be determined. Hence, the aim of this study was to analyze implant osseointegration, bone repair, and sinus mucosa integrity using Bio-Oss® and hyaluronic acid–polynucleotide gel (Regenfast®) in maxillary sinus augmentation in rabbits.

## 2. Materials and Methods

### 2.1. Ethical Considerations

This study was authorized by the Animal Ethics Committee of the Faculty of Dentistry, University of São Paulo (CEUA), Brazil, on 14 June 2023 (protocol #2023.1.342.58.5). This study was conducted in accordance with the National Council for the Control of Animal Experimentation (CONCEA) and fully adhered to the ARRIVE guidelines 2.0 for reporting animal research.

### 2.2. Research Design

Maxillary sinus augmentation was performed bilaterally in rabbits, with two separate osteotomies created per sinus: one anterior and one posterior, according to the protocol described by Martins et al., 2022 [37]. The present study follows a protocol similar to that used in a previous experiment by the same research group [36], although implants were not placed in that earlier study. Consequently, several procedures described in the current Section 2 reflect those previously adopted.

Deproteinized bovine bone mineral (Bio-Oss^®^, Wolhusen, Switzerland) was used as the grafting material at both osteotomy sites. At the test sites (Regenfast^®^, Wolhusen, Switzerland), Bio-Oss^®^ was mixed with a viscoelastic gel, while no gel was applied at the control sites. One implant was placed in the anterior osteotomy of each sinus (implant site), whereas the posterior osteotomies received only the graft material (only grafted sites). Biopsies were collected at 2 and 10 weeks postoperatively, with 6 animals assigned to each healing period.

### 2.3. Animals Used

Twelve adult female New Zealand white rabbits, aged 5–6 months and weighing approximately 3.5–4.0 kg, were included in this prospective, randomized, within-subject study. All animals were obtained from an authorized animal farm for research purposes (ANILAB, Paulínia, São Paulo, Brazil).

### 2.4. Sample Size Calculation

As no preliminary data were available for sample size estimation, it was anticipated that a 5 ± 2.5% difference in new bone formation across the total area after 10 weeks would provide sufficient justification for the treatment. Using a two-tailed test with α = 0.05 and a power of 0.9, the calculated sample size was 5 animal pairs to reject the null hypothesis of no significant difference (PS Power and Sample Size Calculations) [38]. To mitigate any potential issues with the experiment, the sample size was increased to 6 animals.

### 2.5. Randomization and Allocation Concealment

Randomization and allocation concealment were performed through an online system (https://randomizer.org, accessed on 4 March 2024) by an investigator (S.L.S.d.S.) who had no involvement in animal selection, surgical procedures, or outcome assessment. The treatment group assignments were enclosed in sealed, opaque envelopes, which remained unopened until the moment of graft placement. Blinding was ensured for both histological and micro-CT analyses; evaluators were unaware of the assigned healing period and treatment type, and histological samples were anonymized through coded labeling.

### 2.6. Implant Characteristics

The implants used in the study were custom designed for the research by S.I.N.^®^ (S.I.N. Implant System, São Paulo, Brazil). These titanium implants had a diameter of 3 mm and a length of 4 mm, featuring an external thread design and a milling process that promoted high stability and minimized compression of the surrounding bone. Furthermore, the implants were treated with a surface coating of hydroxyapatite nanocrystals [39,40,41].

### 2.7. Biomaterials Used

Bio-Oss^®^ (Bio-Oss^®^, Geistlich, Switzerland) is a xenogeneic bone substitute that is osteoconductive and slowly resorbed. It is derived from the mineral portion of bovine bone, which undergoes deproteinization to yield a highly porous, inorganic structure that closely resembles human cancellous bone in its physical and chemical properties [42].

Regenfast^®^ is a product that combines hyaluronic acid with highly purified, fully resorbable polynucleotides, designed to assist in the regeneration of oral tissues [33]. When combined with a particulate xenogeneic graft, it forms a pasty material.

### 2.8. Anesthetic Protocol

Anesthesia was induced via intramuscular injection of a combination of acepromazine (1.0 mg/kg; Acepran, Vetnil, Louveira, Brazil), xylazine (3.0 mg/kg; Dopaser^®^, Hertape Calier, Juatuba, Brazil), and ketamine hydrochloride (50.0 mg/kg; União Química Farmacêutica Nacional S/A, Embu-Guaçu, Brazil). Following sedation, animals received prophylactic oxytetracycline (0.2 mL/kg; Biovet, Vargem Grande Paulista, Brazil); subcutaneous meloxicam, 0.2% (1.0 mg/kg; Flamavet, União Química Farmacêutica S/A. Embu-Guaçu, Brazil); and tramadol hydrochloride (5.0 mg/kg; Halexistar, Goiânia, Brazil). The surgical field was shaved and disinfected using a 1% iodine-based polyvinylpyrrolidone solution (Riodeíne Tincture, Rioquímica, São José do Rio Preto, Brazil). Local infiltration anesthesia was performed with 2% mepivacaine containing 1:100,000 norepinephrine (Mepinor, Nova DFL, Rio de Janeiro, Brazil).

### 2.9. Surgical Procedures

**Figure 1:**
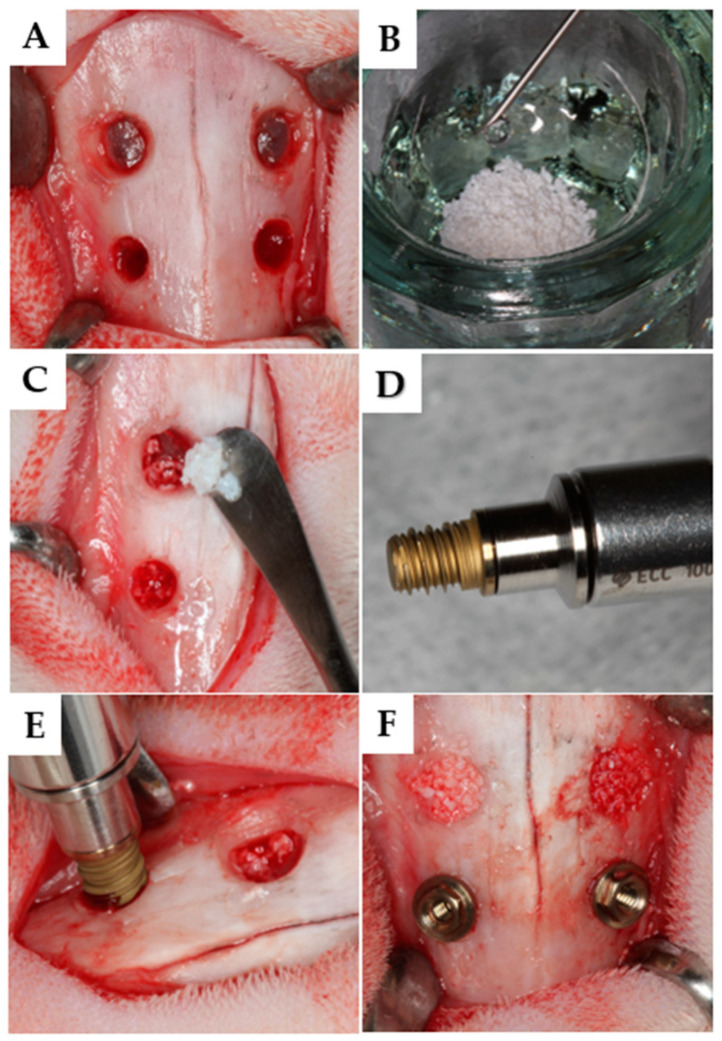
Clinical procedures. (**A**) Preparation of the osteotomies laterally to the naso-incisal suture. (**B**) Bio-Oss® granules were dispensed into a glass dish using a measuring spoon. Regenfast® was added and mixed to the xenograft. (**C**) After sinus mucosa elevation, the graft was inserted into the elevated space. (**D**) In this study, 3 mm × 4 mm implants (S.I.N.® Implant System) were used. (**E**) The implants were installed in each osteotomy at bone level. (**F**) Final trans-operative view.

The surgeries were performed by a single, highly experienced operator (V.F.B.; see acknowledgments). A 2.0 cm incision was made along the nasal dorsum’s midline, followed by careful dissection through muscle layers to expose the periosteum and nasal bone (Figure 1A).

One circular window, 5.5 mm in diameter, was created using a trephine drill (S.I.N. Implant System, São Paulo, Brazil) anteriorly to the nasofrontal suture and about 4 mm lateral to the naso-incisal suture, bilaterally (only grafted sites). Subsequently, osteotomies for implant placement (implant sites) were prepared anteriorly to the first windows using a sequence of drills as recommended by the manufacturer (S.I.N. Implant System, São Paulo, Brazil). The sinus mucosa was elevated according to previously described techniques [37,43].

Following the established randomization, the elevated maxillary sinus on the control side was filled with 2 cc (100 mm^3^) of Bio-Oss^®^ small granules, 0.25–1 mm in size (Geistlich, Wolhusen, Switzerland), through the two osteotomies (Figure 1B,C). On the test side, a mixture of Regenfast^®^ (0.1 mL, Mastelli, Sanremo, Italy) and Bio-Oss^®^ Small granules was used, with the total volume being the same as on the control side. Specially designed implants, 3 mm in diameter and 4 mm in length (Figure 1D), were placed manually into the anterior osteotomies (Figure 1F), until the cervical portion was leveled with the bone (Figure 1F). The periosteum was closed with resorbable sutures (Polyglactin 910 5-0, Vicryl^®^, Ethicon, Johnson & Johnson, São José dos Campos, Brazil), and the skin was sutured with nylon sutures (Ethilon 4-0^®^, Ethicon).

### 2.10. Animal Care

Postoperative care included the administration of anti-inflammatory and analgesic agents for the first three days following surgery. Meloxicam 0.2% (0.5 mg/kg; Flamavet, União Química Farmacêutica S/A) and tramadol hydrochloride (5.0 mg/kg; Halexistar) were delivered subcutaneously to manage pain and inflammation.

The animals were housed individually in standard metal cages (surface area: 4500 cm^2^ per rabbit) within the Animal Facility of the Faculty of Dentistry at the University of São Paulo, Ribeirão Preto campus. Environmental conditions were carefully controlled, with ambient temperatures maintained between 20 and 22 °C, relative humidity around 50%, and 27–34 air changes per hour ensured via air conditioning and exhaust systems. A 12-h light/dark cycle was implemented. The rabbits received a specialized diet and had ad libitum access to water. Throughout the experimental period, a rigorous monitoring protocol was adopted, involving daily evaluation of general health status, feeding behavior, excretory function, and signs of discomfort or postoperative pain. Surgical sites were inspected for wound integrity, bleeding, infection, or suture dehiscence.

### 2.11. Euthanasia

Euthanasia was carried out at the predetermined time points of 2 and 10 weeks, based on the established randomization protocol. Initial sedation was achieved via intramuscular injection of acepromazine (1.0 mg/kg; Acepran^®^, Vetnil), xylazine (3.0 mg/kg; Dopaser^®^, Hertape Calier), and ketamine hydrochloride (50 mg/kg; Ketamin Agener, União Química Farmacêutica S/A). Following sedation, the animals were placed in a carbon dioxide chamber with a regulated gas flow of 7 L/min, corresponding to 20% of the chamber volume. Gas flow was sustained for at least one additional minute after confirmation of clinical death, which was determined by the cessation of respiration, cyanosis of the mucosa, and the absence of palpable pulse.

### 2.12. Tissue Processing

The specimens underwent dehydration through a graded ethanol series (from 50% to 100%) over a period of six days. Subsequently, they were infiltrated with increasing concentrations of resin (LR White™ HardGrid, London Resin Co Ltd., Berkshire, UK), ranging from 50% to 100%. Polymerization was carried out in an oven at 60 °C for 24 h. The resin-embedded blocks were then sagittally sectioned, with the implant axis serving as the reference. Sections measuring approximately 100–150 μm were obtained using a precision cutting system (Exakt, Apparatebau, Norderstedt, Germany), and further ground down to a final thickness of 50–60 μm. Histological staining was performed using protocols recently refined by our group, including toluidine blue, Stevenel’s blue, and alizarin red.

### 2.13. Histomorphometric Analysis

Histological analysis was conducted by a calibrated examiner (E.R.S.) under the supervision of an experienced histologist (S.P.X.). Inter-examiner agreement for the identification of histological structures was high, with a kappa coefficient exceeding 0.90. Observations were made using a light microscope (Leica Microsystems, Bensheim, Germany) equipped with a digital camera (Leica DC 300F, Bensheim, Germany) and connected to a computer for image capture and analysis. Quantitative assessments were performed using Image J software (version 1.50i; National Institutes of Health, Bethesda, MD, USA). Tissue composition was determined using a point-counting method, applying an 80-square grid over each histological section at 100× magnification.

**Figure 2:**
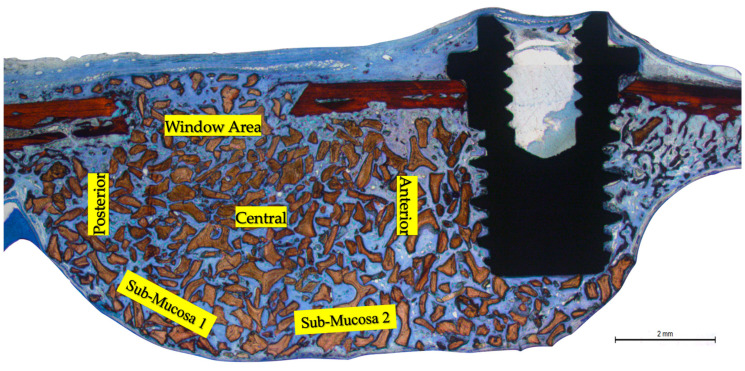
Photomicrograph of ground section illustrating the six areas evaluated: sub-mucosa (two areas); central; sub-window; medial and lateral walls. Stevenel’s blue and Alizarin Red staining.

Morphometric evaluation was limited to grafted areas only. The percentages of new bone, residual xenograft, and soft tissue were assessed across distinct regions: the submucosal area (divided into two zones), the medial and lateral sinus walls, the central compartment, and the sub-window region (Figure 2).

**Figure 3:**
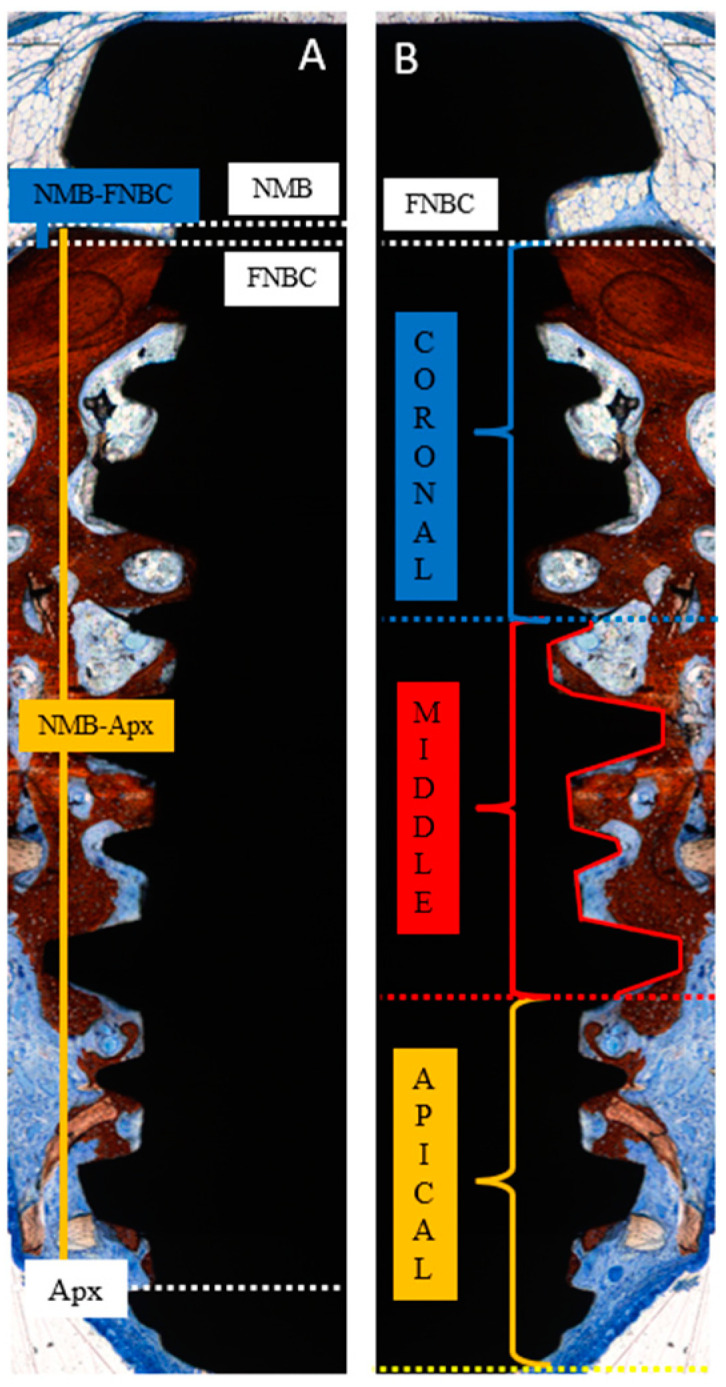
Landmarks for histomorphometric analysis. (**A**) Linear measurements indicating the distances between the native marginal bone (NMB) to the coronal level of osseointegration (first newly formed bone contact; FNBC) and between NMB and the apical level of osseointegration (Apx). (**B**) Bone-to-implant percentage in three implant areas: coronal, medial and apical. Stevenel’s blue and alizarin red staining.

At the implant sites, two linear measurements were performed as follows (Figure 3A):

1. NMB-FNBC: the distance from the native marginal bone (NMB) to the first newly formed bone contact (FNBC) at the most coronal point of osseointegration.
2. NMB-Apx: the distance from the native marginal bone (NMB) to the most apical point of osseointegration (Apx).

Bone-to-implant contact (BIC) was evaluated in three distinct regions of equal length: coronal, middle, and apical (Figure 3B).

The width of the pristine (non-elevated) sinus mucosa, as well as the number and width of thinned mucosal sites (defined as regions with a width < 40 µm) in contact with graft granules, were evaluated. In addition, the number and dimensions of mucosal perforations were assessed. The width of the pseudostratified epithelium was also measured in both pristine and thinned mucosal areas.

### 2.14. Statistical Analysis

The results are presented as mean ± standard deviation. The following primary variables were considered: new bone percentage for the only grafted sites; NMB-Apx (that is the extension of osseointegration along the implant) for the implant sites; the number of perforations for the sinus mucosa. The Shapiro–Wilk test was used to assess normality. Based on these results, differences between test and control groups were analyzed using either a paired *t*-test or a Wilcoxon matched-pairs signed-rank test, while differences between periods were analyzed applying an unpaired *t*-test or a Mann–Whitney test. Statistical analyses were performed with GraphPad Prism (version 10 for Windows, GraphPad Software, Boston, MA, USA), with a significance level set at 5%.

## 3. Results

### 3.1. Clinical Outcomes

The healing of the animals was uneventful. All histological slides were available for analysis, with *n* = 6 for both periods.

### 3.2. Histological Evaluation at the Only Grafted Sites

**Figure 4:**
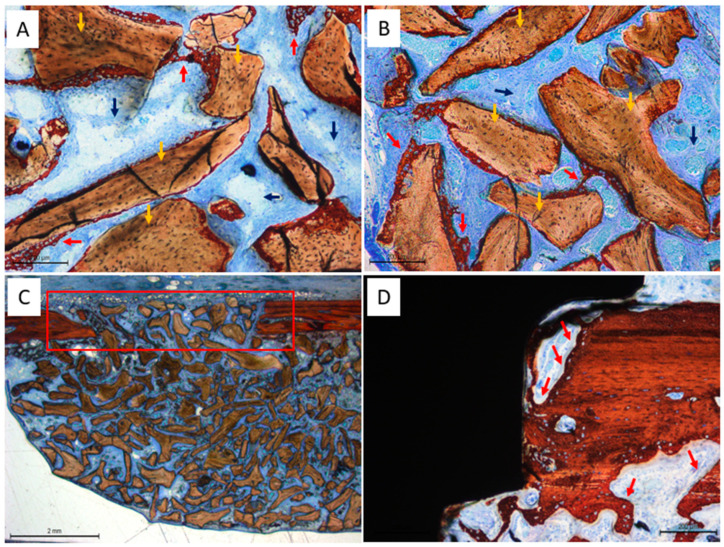
Photomicrographs of ground sections after 2 weeks of healing. The elevated area was predominantly occupied by xenograft particles and soft tissue both in the test (**A**) and control sites (**B**). (**C**) Photomicrograph showing the open osteotomy window after two weeks of healing. (**D**) New bone formation observed from the adjacent cortical bone layers. Yellow arrows indicate residual graft material, while red arrows highlight areas of new bone formation. The red square outlines the antrostomy site. Stevenel’s blue and alizarin red staining.

After two weeks of healing (Table 1), the elevated area was predominantly occupied by xenograft particles and soft tissue, with less than 10% of new bone observed across all evaluated regions in both the test sites (treated with Regenfast^®^; Figure 4A) and the control sites (Figure 4B). The osteotomy windows remained open in all cases (Figure 4C); however, new bone formation was frequently observed originating from the adjacent cortical bone layers (Figure 4D).

**Figure 5:**
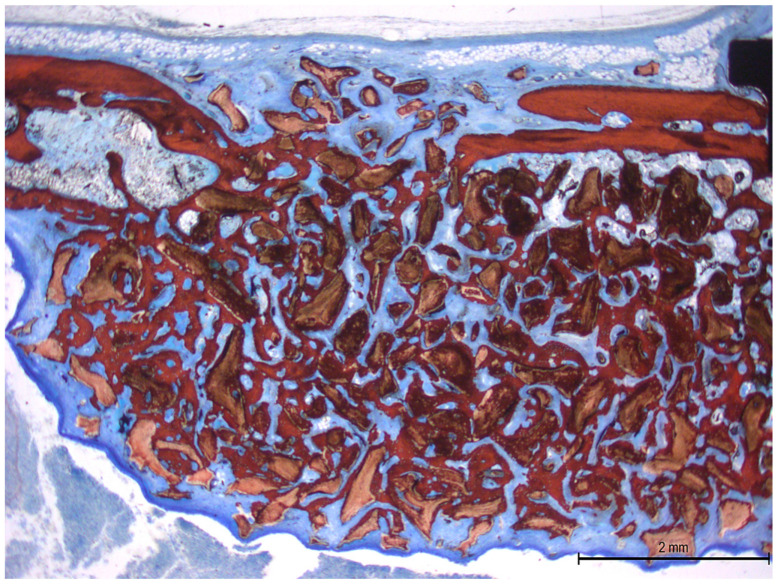
Photomicrograph of ground section indicating an increase in the fraction of new bone after ten weeks of healing, compared to the earlier time point, and an initial corticalization of the osteotomy. Stevenel’s blue and alizarin red staining.

After ten weeks of healing (Table 2), the fraction of new bone increased in all evaluated regions compared to the earlier time point (Figure 5), with higher values observed in the test sites compared to the controls (*p* = 0.031; effect size r ≈ 0.88). Among the parameters evaluated, only the total amount of new bone showed a statistically significant difference between groups (*p* = 0.031). A corticalization of the osteotomy was often observed (Figure 5).

### 3.3. Histological Evaluation at the Implant Sites

**Figure 6:**
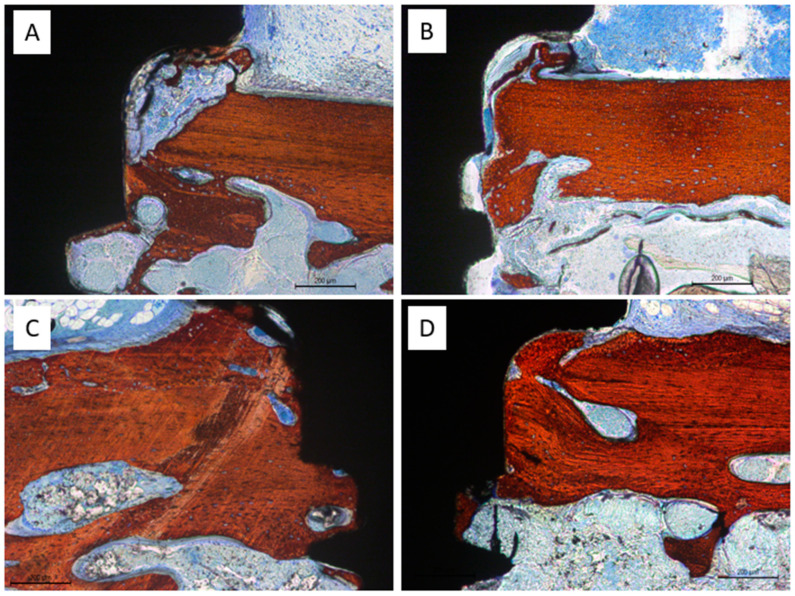
Photomicrographs of ground sections indicating a limited amount of marginal bone resorption in two weeks of healing at both control (**A**) and test (**B**) sides, and in ten weeks of healing for control (**C**) and test (**D**) sides. Stevenel’s blue and alizarin red staining.

**Figure 7:**
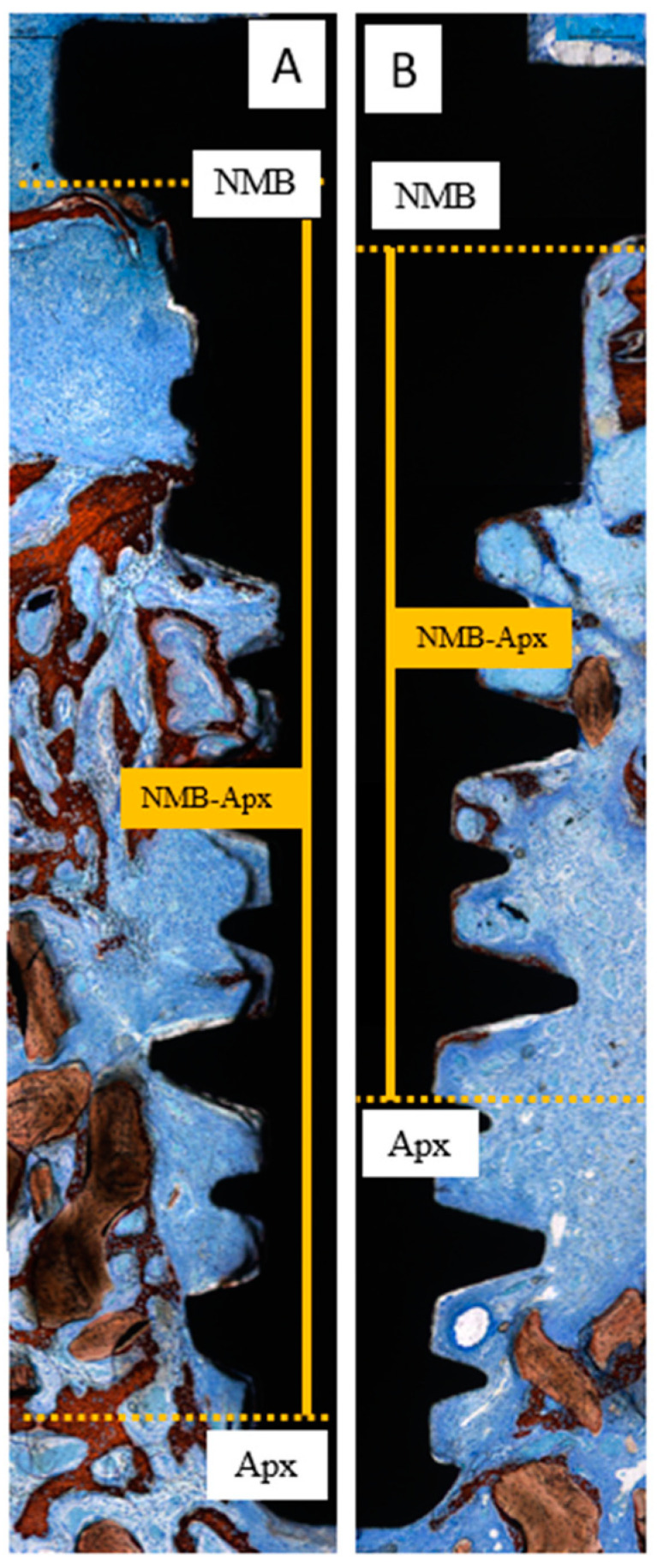
Photomicrographs of ground sections illustrating the differences in the mean apical extension of osseointegration from native marginal bone (NMB-Apx) in the control (**A**) and test (**B**) sides in two weeks of healing. Stevenel’s blue and alizarin red staining.

**Figure 8:**
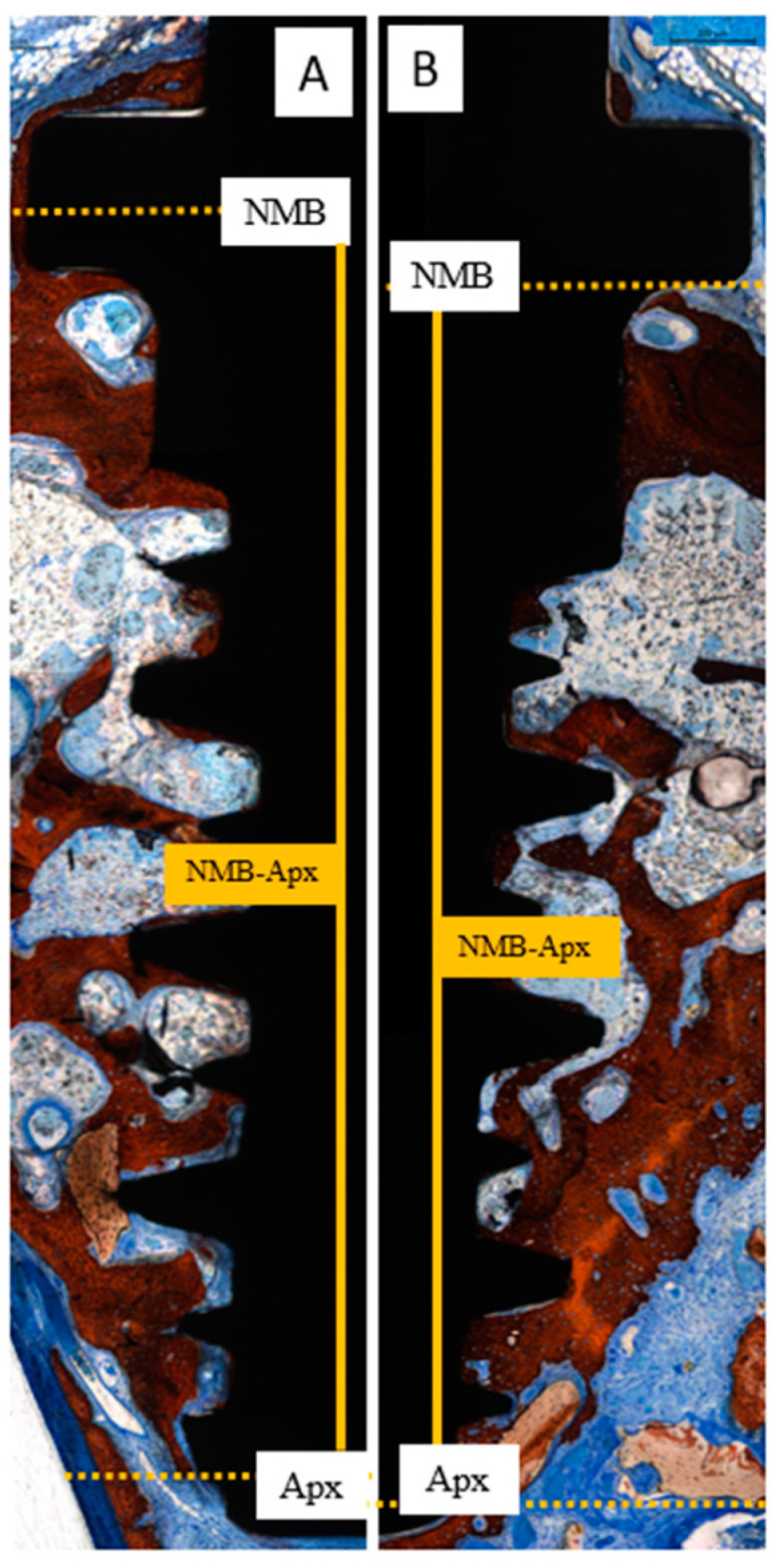
Photomicrograph of ground sections indicating that in ten weeks of healing the apical extension of native marginal bone (NMB-Apx) became equivalent in both control (**A**) and test groups (**B**). Stevenel’s blue and alizarin red staining.

A limited amount of marginal bone resorption was observed at both the test and control sites at both healing intervals (Figure 6). After 2 weeks of healing, the mean apical extension of osseointegration from the newly formed marginal bone (NMB-Apx) was 3.68 ± 1.10 mm in the control sites and 2.62 ± 1.14 mm in the test sites (Table 3; Figure 7), with a statistically significant difference (*p* = 0.031; effect size r ≈ 0.88). However, after 10 weeks of healing, osseointegration had reached the apex of the implant in both groups (*p* = 0.604; Table 3; Figure 8).

At 2 weeks, the overall bone-to-implant contact (BIC%) was 16.1 ± 8.2% in the control group and 17.9 ± 8.8% in the test group (*p* = 0.350). The highest BIC% values were found in the coronal regions, while the lowest were observed apically. Osseointegration percentages increased markedly in both groups across all evaluated regions after 10 weeks of healing, with no statistical differences (Table 4).

### 3.4. Mucosa Assessments

**Figure 9:**
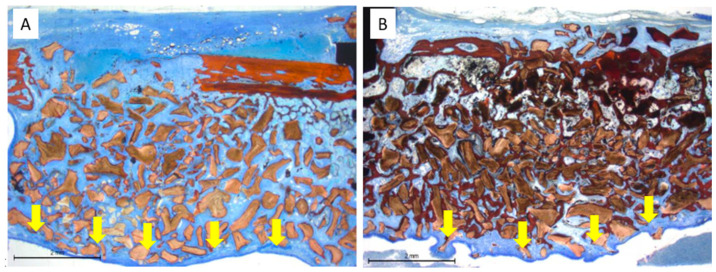
The yellow arrows indicate the sinus mucosa pattern. (**A**) Smooth mucosa in two weeks of healing. (**B**) Corrugated configuration following the contours of adjacent granules. A rectangle indicates the specific area of interest within the histological section. Stevenel’s blue and Alizarin Red staining.

Mucosal modifications, including thinning and perforation, were frequently observed in association with deproteinized bovine bone mineral (Bio-Oss^®^) granules extending beyond the dome-shaped profile of the sinus augmentation. This was particularly evident at the 10-week time point, where the mucosa exhibited a corrugated configuration, with ridges and peaks closely conforming to the contours of adjacent granules (Figure 9).

**Figure 10:**
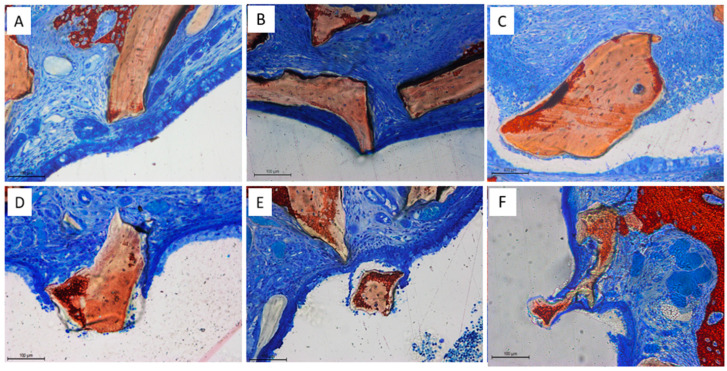
(**A**) Photomicrograph showing thinned epithelium limited to the lamina propria, with blood vessels and mucosal glands near the xenograft. (**B**) Epithelial thinning became evident, with reduction of goblet cells and cilia. (**C**) Connective tissue layer remnant between granules and the sinus cavity in most severe cases. (**D**) Epithelial perforation associated with angular granule surface. (**E**) Reparative response, with signs of displacement of granules from the augmented space. (**F**) Tapered morphology of the surrounding epithelium, indicating the attempt to re-establish separation from the sinus lumen. Stevenel’s blue and alizarin red staining.

At both control and test sites, and across all healing intervals, the sinus mucosa presented multiple areas of reduced thickness in close proximity to the graft particles. These alterations appeared to follow a progression. Initially, thinning was limited to the lamina propria, with displacement of blood vessels and mucosal glands (Figure 10A). In more advanced cases, epithelial thinning became evident, accompanied by a reduction in goblet cells and cilia (Figure 10B). In the most severe stages, only a thin epithelial or connective tissue layer remained between the granules and the sinus cavity (Figure 10C). Epithelial perforations were eventually noted, particularly in contact with sharp or angular granule surfaces (Figure 10D). Reparative responses were also observed, with epithelial and connective tissues attempting to restore the integrity of the mucosa, along with signs of encapsulation or displacement of granules from the augmented space (Figure 10E). The surrounding epithelium often displayed a tapered morphology around granules, potentially indicating an effort to reestablish separation from the sinus lumen (Figure 10F).

Quantitative analysis confirmed these observations. The thickness of the pristine sinus mucosa ranged from 97 to 122 µm on average (Table 5). At the 2-week time point, 31 thinned sites (<40 µm) were identified in the control group, with a mean thickness of 16 ± 11 µm, and 27 in the test group, averaging 21 ± 11 µm. In both groups, the thinnest mucosa measured 4 µm. Notably, seven sites in the control group exhibited a thickness <10 µm, compared to only one site in the test group. Two perforations, each affecting a different sinus, were observed in the control group, while a single perforation occurred in the test group.

After 10 weeks of healing (Table 5), the number of thinned mucosa sites more than doubled in both groups compared to the 2-week period, and the pseudostratified epithelium at these sites demonstrated further thinning. Perforation frequency also increased, with fourteen events recorded in the control group (across six sinuses) and five in the test group (across two sinuses). The mean width of the epithelial cells in unaffected regions ranged from 27 to 35 µm, while in thinned mucosa areas, the cell width was reduced to between 13 and 16 µm.

## 4. Discussion

In the present study, a product combining hyaluronic acid with highly purified, fully resorbable polynucleotides (Regenfast^®^), mixed with deproteinized bovine bone mineral (Bio-Oss ^®^), was used for maxillary sinus floor elevation in rabbits. Two different regions were evaluated, one with an implant installed simultaneously at the surgery (implant site) and one without any implant (only grafted region). In the only grafted site, after 10 weeks of healing, in the total region a higher new bone percentage was observed at the test (Regenfast^®^) sites compared to the control sites (*p* = 0.031; effect size r ≈ 0.88). No other statistically significant differences were observed for all other variables evaluated. In the implant sites, after 2 weeks of healing, a higher apical migration of osseointegration was observed in the control sites compared to the test (Regenfast^®^) sites (*p* = 0.031; effect size r ≈ 0.88).

The results observed in the only grafted region differ from those obtained in a previous study conducted by the same research group, in which no differences in new bone formation were found between the groups. In that experiment, sinus floor elevation was also performed bilaterally in rabbits, using either (Bio-Oss ^®^) alone or combined with Regenfast^®^, but only one osteotomy per side was made, and no implants were inserted. After 2 and 10 weeks of healing, similar amounts of new bone were detected in both groups.

A key methodological difference between the two studies lies in the histological sectioning technique. In the previous study [36], sections were cut in the coronal plane, which included the lateral and medial sinus walls, areas with high osteogenic potential and known to be the main sources of new bone formation [4,19]. In that environment, the greatest amounts of new bone were observed near these walls after 2 weeks, while the central and sub-mucosa zones showed minimal bone content.

In contrast, the current study employed sagittal sections, focusing on the central and sub-mucosa regions, areas that typically exhibit delayed bone formation and are less influenced by direct osteogenic sources [4,19]. After 10 weeks of healing, the control group exhibited a similar amount of new bone to the previous study [36]. However, Regenfast^®^ showed a more pronounced effect: a significantly greater total amount of new bone was observed in the test group compared to the control, with increased bone formation across all regions, although statistical significance was reached only for the overall amount.

This suggests that the regenerative effect of Regenfast^®^ may be more detectable in areas with limited spontaneous bone formation, such as the central and sub-mucosa regions. Additionally, although the implants were not located in the analyzed regions, their simultaneous placement may have contributed to an overall more active healing environment.

Nevertheless, while Regenfast^®^ showed a favorable effect on bone formation, that effect on osseointegration was inferior. In fact, after 2 weeks, the extension of osseointegration was higher at the control compared to the test sites and did not reach the apex of the implant. After 10 weeks of healing, osseointegration reached the apex in both groups. The pattern of healing showed that integration started from the base of the implant, that is, from the cortical bone layer, proceeding towards the apex of the implant. This pattern of healing was already described in a similar study both in rabbits [44] and at immediate implants [45]. This pattern of healing implies an important consideration that is based on the osteoconductivity of the implant surface. In fact, when a turned surface was used in marginal defect, the healing was incomplete [46].

An additional noteworthy finding was the identification of a sinus mucosa exhibiting a wrinkled or undulating pattern, with elevations and depressions that mirrored the contours of the adjacent graft granules. This pattern was associated with multiple areas of reduced mucosal thickness in direct contact with the graft particles, as well as with several perforations. The phenomenon exhibited a progressive nature, similar to what was first reported in a rabbit study [47]: in the early stages, displacement of glands and blood vessels was observed, followed by gradual thinning of the mucosa and cellular layers, loss of cilia, and ultimately, mucosal perforations. This progressive pattern was further supported by the increasing number of thinning areas and perforations observed over time at both test and control sites. The inability of Regenfast^®^ to protect the sinus mucosa from this process had already been demonstrated in a previous, similar study [36].

Two healing periods were analyzed in the present study to address different objectives: to assess the potential time-dependent effect of Regenfast, the progression of implant osseointegration, and the temporal pattern of sinus mucosa damage caused by the xenograft granules. The results suggested that Regenfast may exert a greater effect between 2 and 10 weeks, osseointegration tends to progress from the coronal aspect toward the apex, and mucosal damage induced by the granules appears to increase progressively over time.

This study has inherent limitations, primarily due to the use of an animal model and the small number of samples, which aligns with ethical guidelines promoting reduction in animal testing. Moreover, translating these results to human clinical settings should be carried out with caution, as the experimental conditions do not fully reflect the biological and systemic variability observed in patients. The standardized and controlled nature of pre-clinical environments, while useful for isolating specific effects, may overlook the multi-factorial influences present in everyday clinical practice. Additionally, the relatively short observation periods typical of animal studies do not permit conclusions regarding the long-term behavior and clinical success of the applied regenerative approach. Therefore, the findings presented here should be interpreted as preliminary and applicable only to similar experimental conditions and short-term healing contexts.

## 5. Conclusions

The addition of Regenfast^®^ to Bio-Oss^®^ granules promoted enhanced bone regeneration within the elevated sinus area and was associated with a lower incidence of sinus membrane perforations compared to the use of Bio-Oss^®^ alone.

## Figures and Tables

**Table 1:** Histomorphometric measurements in the various areas analyzed after 2 weeks of healing. Mean percentage values ± standard deviation.

	Control			Test (Regenfast^®^)		
	New Bone	Xenograft	Soft Tissue	New Bone	Xenograft	Soft Tissue
Total	7.3 ± 2.4 ^‡^	41.6 ± 8.1	51.1 ± 8.6	7.7 ± 2.4 ^‡^	37.9 ± 9.7	54.3 ± 11.3
Sub-Mucosa	8.5 ± 4.6 ^‡^	40.0 ± 12.3	51.5 ± 12.0	8.2 ± 3.2 ^‡^	40.1 ± 11.4	51.7 ± 11.2
Anterior	7.2 ± 4.5 ^‡^	37.6 ± 13.7	55.3 ± 15.2	5.5 ± 4.7 ^‡^	33.1 ± 9.3	61.4 ± 11.0
Central	5.8 ± 4.1 ^‡^	45.6 ± 19.1	48.6 ± 16.2	6.5 ± 4.4 ^‡^	41.5 ± 10.3	51.9 ± 12.1
Posterior	9.0 ± 5.5 ^‡^	39.7 ± 7.8 *	51.3 ± 11.1	12.8 ± 6.4 ^‡^	32.7 ± 10.4 *	54.5 ± 13.1
Sub-Window	5.0 ± 4.3 ^‡^	46.4 ± 13.7	48.6 ± 10.2	5.0 ± 5.6 ^‡^	40.1 ± 13.1	54.9 ± 15.4

*, *p* < 0.05 between test and control (paired *t*-test or Wilcoxon matched-pairs signed-rank test). ^‡^, *p* < 0.05 between periods (unpaired *t*-test or Mann–Whitney test).

**Table 2:** Histomorphometric measurements in the various areas analyzed after 10 weeks of healing. Mean percentage values ± standard deviation.

	Control			Test (Regenfast^®^)		
	New Bone	Xenograft	Soft Tissue	New Bone	Xenograft	Soft Tissue
Total	26.8 ± 10.0 *^‡^	37.1 ± 7.3 ^‡^	36.2 ± 8.8	37.2 ± 6.7 *^‡^	29.7 ± 6.3 ^‡^	33.2 ± 3.3
Sub-mucosa	27.6 ± 15.0 ^‡^	36.5 ± 8.1 ^‡^	36.0 ± 8.4	38.1 ± 12.1 ^‡^	29.9 ± 7.6 ^‡^	31.9 ± 5.9
Anterior	30.1 ± 16.3 ^‡^	31.9 ± 14.3	37.9 ± 15.1	42.9 ± 10.9 ^‡^	25.9 ± 9.2 ^‡^	31.2 ± 4.1
Central	22.9 ± 11.8 ^‡^	39.1 ± 8.4	37.9 ± 9.6	31.3 ± 14.6 ^‡^	39.9 ± 15.1 ^‡^	28.8 ± 2.9
Posterior	29.9 ± 12.2 ^‡^	37.8 ± 9.3 ^‡^	32.3 ± 16.8	40.8 ± 14.3 ^‡^	25.9 ± 16.3 ^‡^	33.3 ± 3.1
Sub-Window	22.7 ± 15.8 ^‡^	40.5 ± 9.5	36.8 ± 14.8	31.8 ± 15.8 ^‡^	26.4 ± 9.6	41.8 ± 12.4

*, *p* < 0.05 between test and control (paired *t*-test or Wilcoxon matched-pairs signed-rank test). ^‡^, *p* < 0.05 between periods (unpaired *t*-test or Mann–Whitney test).

**Table 3:** Histological measurements of the distance between the native marginal bone (NMB) and the most apical extension of osseointegration (Apx) and the first newly formed bone contact (FNBC). Data expressed in millimeters.

	Two Weeks		Ten Weeks	
	NMB-Apx	NMB-B	NMB-Apx	NMB-B
Control	3.68 ± 1.10 *	0.49 ± 0.65 ^‡^	3.73 ± 0.27	0.04 ± 0.05 ^‡^
Test (Regenfast^®^)	2.62 ± 1.14 *	0.20 ± 0.12	3.84 ± 0.25	0.36 ± 0.51
*p*-value	0.031	0.300	0.604	0.625

*, *p* < 0.05 between test and control (paired *t*-test or Wilcoxon matched-pairs signed-rank test). ^‡^, *p* < 0.05 between periods (unpaired *t*-test or Mann–Whitney test).

**Table 4:** Histological measurements of bone-to-implant contact (BIC) evaluated at different levels of the implants and as total amount.

		Total	Apical	Middle	Coronal
Two weeks	Control	16.1 ± 8.2 ^‡^	5.5 ± 3.4 ^‡^	18.2 ± 15.4 ^‡^	24.4 ± 16.5 ^‡^
	Test (Regenfast^®^)	17.9 ± 8.8 ^‡^	4.0 ± 4.5 ^‡^	15.0 ± 18.2 ^‡^	34.7 ± 7.6
	*p*-value	0.350	0.544	0.602	0.064
Ten weeks	Control	42.3 ± 7.8 ^‡^	29.0 ± 16.0 ^‡^	41.3 ± 10.8 ^‡^	56.7 ± 11.2 ^‡^
	Test (Regenfast^®^)	40.2 ± 9.8 ^‡^	27.2 ± 12.8 ^‡^	48.0 ± 19.1 ^‡^	45.4 ± 23.9
	*p*-value	0.687	0.833	0.352	0.295

*p* < 0.05 between test and control (paired *t*-test or Wilcoxon matched-pairs signed-rank test). ^‡^, *p* < 0.05 between periods (unpaired *t*-test or Mann–Whitney test).

**Table 5:** Data from 2 and 10 weeks of healing. Width of the pristine mucosa; number of sites with mucosal thinning; mean width ± standard deviation; minimum width and number of sites with mucosa <10 µm; number and size of perforations; and number of sinuses involved.

		Pristine µm	Thinned Mucosae				Perforations		
			No	Mean µm	Min µm	<10 µm	No	Dimension µm	Sinus
2 weeks	Control	117 ± 41	31	16 ± 11	4	7	2	311 ± 76	2
	Test	115 ± 50	27	21 ± 11	4	1	1	2005	1
10 weeks	Control	97 ± 33	64	22 ± 4	3	12	14	337 ± 486	6
	Test	122 ± 38	62	23 ± 5	1	15	5	1522 ± 2095	2

## Data Availability

The original contributions presented in the study are included in the article; further inquiries can be directed to the corresponding author.

## Abbreviations

The following abbreviations are used in this manuscript:

HyA:	Hyaluronic acid
PN:	Polynucleotides
NMB:	Native marginal bone
FNBC:	First newly formed bone contact
Apx	Apical extension of osseointegration
